# Effect of Adjuvants on Herbicidal Activity and Selectivity of Three Phytotoxins Produced by the Fungus, *Stagonospora* *cirsii*

**DOI:** 10.3390/plants9111621

**Published:** 2020-11-21

**Authors:** Vsevolod Dubovik, Anna Dalinova, Alexander Berestetskiy

**Affiliations:** All-Russian Institute of Plant Protection, Podbelskogo St., 3, Pushkin, Saint-Petersburg 196608, Russia; xasevak@gmail.com (V.D.); adalinova@vizr.spb.ru (A.D.)

**Keywords:** phytotoxin, nonenolide, natural product-derived herbicide, *Stagonospora cirsii*, stagonolide A, stagonolide K, herbarumin I, adjuvant

## Abstract

The use of many fungal phytotoxins as natural herbicides is still limited because they cannot penetrate leaf cuticle without injury and a little is known on their selectivity. In order to assess the herbicidal potential of phytotoxic 10-membered lactones (stagonolide A, stagonolide K, and herbarumin I), the selection of adjuvants, the evaluation of selectivity of the toxins and the efficacy of their formulations were performed. Among four adjuvants tested, Hasten™ (0.1%, *v*/*v*) increased phytotoxic activity of all the toxins assayed on non-punctured leaf discs of *Sonchus arvensis*. When assayed on intact leaf fragments of 18 plants species, 10 species were low to moderately sensitive to stagonolide A, while just five and three species were sensitive to stagonolide K and herbarumin I, respectively. Both leaf damage or addition of Hasten™ (0.1%) to the formulations of the compounds considerably increased or altered the sensitivity of plants to the toxins. Stagonolide A was shown to be non-selective phytotoxin. The selectivity profile of stagonolide K and herbarumin I depended on the leaf wounding or the adjuvant addition. Stagonolide A and herbarumin I formulated in 0.5% Hasten™ showed considerable herbicidal effect on *S. arvensis* aerial shoots. This study supported the potential of the oil-based adjuvant Hasten™ to increase the herbicidal efficacy of natural phytotoxins.

## 1. Introduction

The application of chemical control has aided humanity to increase crop productivity for many years, but over the past few decades the intensive use of synthetic herbicides has led to non-targeted adverse environmental effects, soil and water contamination and herbicide resistance in weeds [1]. The bioherbicides and natural product-derived herbicides (NPDH) are considered as relatively safe alternatives for weed control in both organic and conventional agriculture [2,3].

Natural phytotoxins of microbial and plant origin have been used as base scaffolds for new NPDHs. Glufosinate (a synthetic mixture of d- and l-phosphinothricin, the latter is a breakdown product of natural phytotoxin bialophos isolated from *Streptomyces* spp.), manuka oil (from the plant, *Leptospermum scoparium*) and triketone herbicides (structural analogues of leptospermone isolated from *L. scoparium*) are examples of successful NPDHs [4]. Various phytotoxins isolated from fungi were described in the literature but none of them have been commercialized yet. Among them, several compounds look promising for NPDH development. For instance, ascaulitoxin and its aglycone, phytotoxic non-proteinogenic amino acids produced by *Ascochyta caulina* [5], showed the herbicidal potential as reviewed by Cimmino et al., 2015 [6]. *Phoma macrostoma* produces a number of phytotoxic derivatives of tetramic acid (macrocidins) inhibiting the carotenoid biosynthesis [7,8,9]. Recently, tenuazonic acid, a well-known toxin of *Alternaria* spp., was proved to control some troublesome weeds under the field conditions. This relative success was provided by extensive studies on ecotoxicology, action mechanisms, herbicidal selectivity of the toxin, as well as selection of its appropriate formulation [10,11].

The development of NPDHs based on many other fungal phytotoxins is delayed because little is known about their selectivity and general toxicity. The poor selectivity of natural phytotoxins may limit their potential as plant protection products. It is desirable that NPDHs are effective against weeds and safe for at least some of the major crops. Therefore, phytotoxic selectivity assays of natural compounds should target both main weeds and common crops of the area for their potential application [10,12,13]. Moreover, there are some examples of potent natural phytotoxins (for instance, AAL-toxin) that display non-target toxicity that would preclude them from development as NPDHs [3].

Most natural phytotoxins seem to be unable to penetrate the plant cuticle. For this reason, leaf bioassays for the rapid screening of phytotoxic compounds include the wounding of the leaf surface prior to toxin application (the leaf puncture assay) or vacuum and syringe infiltration. However, these bioassays are not good indicators for herbicidal activity of natural compounds in vivo [5,14,15,16,17,18,19]. In the case of chemical herbicides, the problem of their effective absorption into plant tissues is often solved by supplementation with the appropriate adjuvants (surfactants, penetrants, etc.). The effectiveness of the foliar-applied herbicides on target weeds is highly affected by the type of adjuvant added into formulation [20,21]. On the other hand, the addition of adjuvants can alter the selectivity of chemical herbicides and NPDHs which in turn can lead to the decrease in crop tolerance [22,23,24,25].

**Figure 1:**
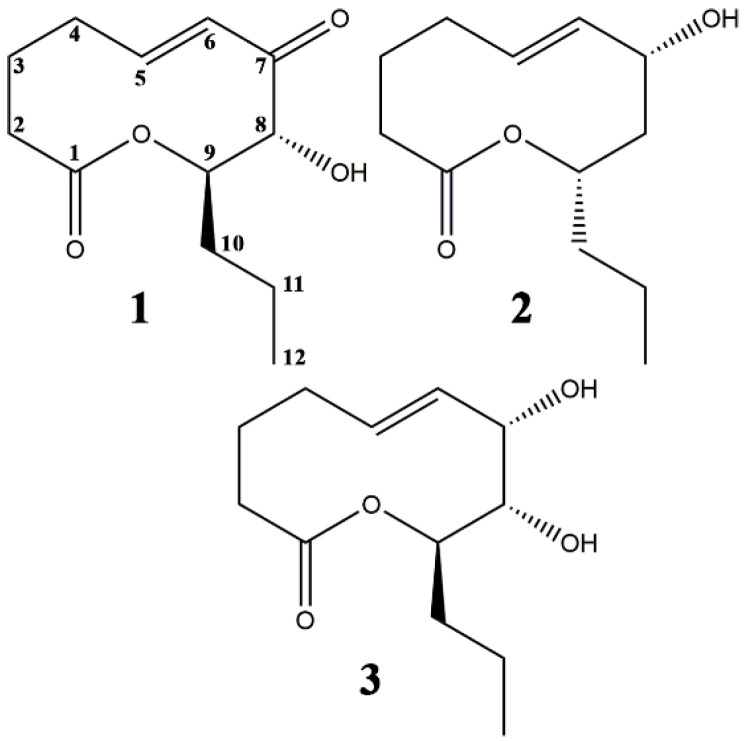
Nonenolide-type phytotoxins from *S. cirsii* S-47. **1**—stagonolide A, **2**—stagonolide K, **3**—herbarumin I.

The fungal secondary metabolites belonging to the nonenolide subgroup of macrolactones (10-membered lactones), for example some putaminoxins, herbarumins, stagonolides and others, are known as promising phytotoxins for NPDH development [26,27,28,29,30]. Three nonenolide phytotoxins, namely stagonolides A and K, and herbarumin I (Figure 1), isolated from *Stagonospora cirsii* S-47, a fungal pathogen of perennial sowthistle (*Sonchus arvensis* L.) [26,30,31,32], were used in this study. All the three toxins were proven to be active against *S. arvensis* in leaf puncture bioassay (the minimum concentrations that caused visible necrosis were 0.25, 2.0 and 1.0 mg/mL for stagonolide A, K and herbarumin I, respectively) [32].

The main objective of this research was to assess the prospects of *S. cirsii* toxins as new post-emergence natural herbicides. The essential steps of the study were (a) the screening of the most compatible adjuvant for foliar application of *S. cirsii* toxins; (b) the evaluation of their phytotoxic selectivity to weeds and crops belonging to different plant families; (c) the assessment of the herbicidal efficacy of *S. cirsii* toxins against perennial sowthistle.

## 2. Results

### 2.1. Effect of Leaf Damage and Adjuvants on Phytotoxicity of S. cirsii Toxins

**Figure 2:**
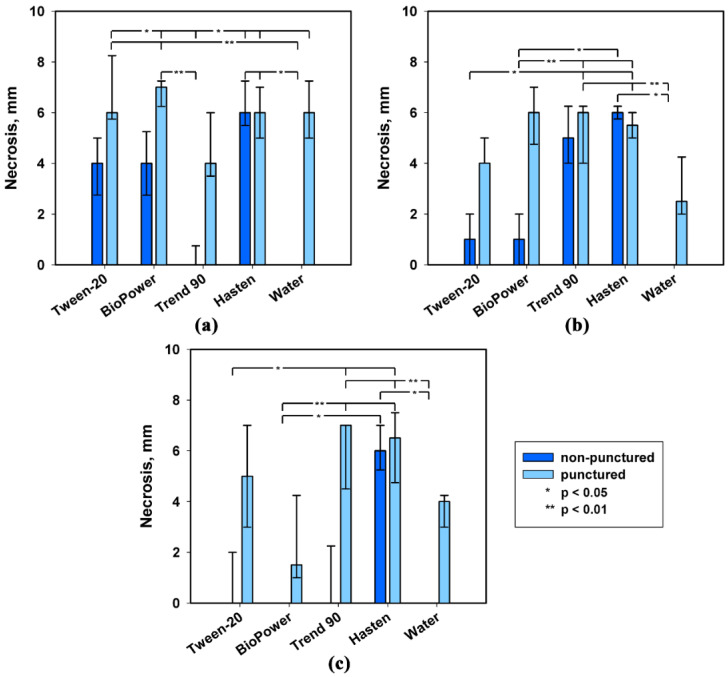
Effect of leaf damage and adjuvants on phytotoxicity of *S. cirsii* S-47 toxins on leaf discs of *S. arvensis*: (**a**) stagonolide A, (**b**) stagonolide K, (**c**) herbarumin I. Bars represent median values and interquartile ranges. Statistically significant differences between groups were assessed by the Kruskal–Wallis test: (*) *p* < 0.05 and (**) *p* < 0.01.

None of the three *S. cirsii* phytotoxins that was not supplemented with adjuvants caused the damage to non-punctured leaf discs of perennial sowthistle at the relatively high concentration of 2 mg/mL, while the punctured leaf discs were sensitive to them. Supplementation of the phytotoxins with five different adjuvants demonstrated their different effects on the development of necrotic lesions on intact and punctured leaf discs of the weed (Figure 2).

The effect of the adjuvant on the phytotoxic activity of stagonolide A was significant (*p* < 0.01) when it was tested on the intact leaf discs. Stagonolide A alone and in the combination with all tested adjuvants caused the development of necrotic lesions on punctured leaf discs and the effect of the adjuvant on the herbicidal activity of the toxin was negligible (Table 1). The phytotoxic activity of stagonolide A with 0.1% Hasten™ on intact leaf discs was at the same level as that on punctured leaves. Tween^®^-20 (0.1% *v*/*v*) and Biopower^®^ (0.1% *v*/*v*) increased sensitivity of intact leaf discs of *S. arvensis* to stagonolide A, but to a lesser extent than Hasten™ (Figure 2a, Table 2).

The use of the adjuvants significantly affected the phytotoxic activity of stagonolide K both on punctured and intact leaf discs of perennial sowthistle (*p* < 0.05 and *p* < 0.001 respectively) (Table 1). The addition of BioPower^®^, Trend^®^-90 (0.01% *v*/*v*), and Hasten™ to the phytotoxin solution increased the activity of stagonolide K on punctured leaf discs compared to the phytotoxin formulation without adjuvant supplementation, while only Trend^®^-90 and Hasten™ were able to provide the penetration of stagonolide K into intact leaves of the weed (Figure 2b).

The supplementation of herbarumin I with the adjuvants had a significant effect on its phytotoxic activity when assayed on both damaged (*p* < 0.05) and intact leaf discs of perennial sowthistle (*p* < 0.001) (Table 1). The combination of herbarumin I with Hasten™ only led to the development of necrotic lesions after the treatment of non-punctured leaf discs of *S. arvensis* (Figure 2c).

The addition of Hasten™ to the liquid formulations of all the tested toxins significantly (*p* < 0.05) increased sensitivity of non-punctured *S. arvensis* leaf discs to them compared to their activity without the adjuvant (Figure 2). Notably, the leaf damage had no effect on phytotoxicity of the nonenolides formulated in 0.1% Hasten™ (Table 2). The phytotoxicity of *S. cirsii* toxins supplemented with Hasten™ on non-punctured leaf discs was relatively high (necrosis diameter about 6 mm) with negligible differences among them, while their activity without adjuvants showed significant differences (*p* < 0.05) (Table 3).

No adjuvant used in this experiment at mentioned concentrations was phytotoxic for non-punctured and punctured leaf discs of *S. arvensis*.

Taking in account positive effect of Hasten™ on phytotoxic activity of all the assayed phytotoxins, this adjuvant was used in further experiments.

### 2.2. Phytotoxic Selectivity of S. cirsii Toxins

**Figure 3:**
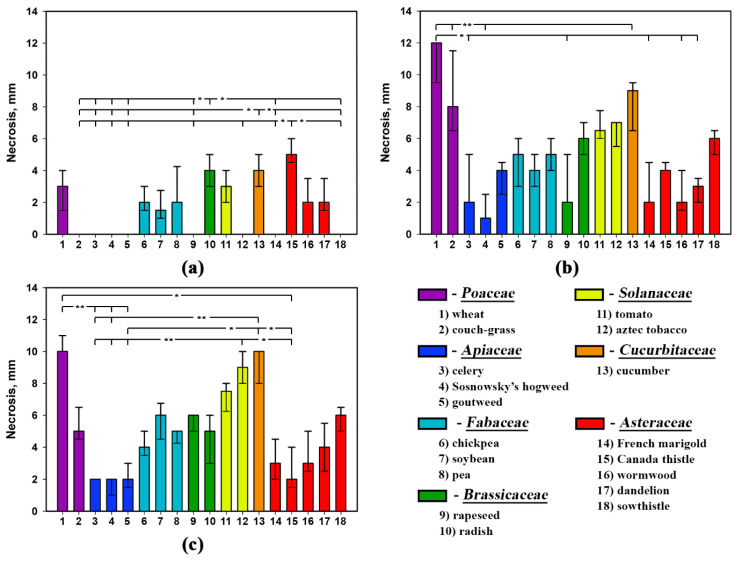
Phytotoxicity of stagonolide A (2 mg/mL) prepared in 5% EtOH assayed on (**a**) non-punctured and (**b**) punctured leaf fragments, and (**c**) supplemented with Hasten™ (0.1% *v*/*v*) on non-punctured leaf fragments of 18 different plant species. Bars represent median values and interquartile ranges. Statistically significant differences between groups were assessed by the Kruskal–Wallis test: (*) *p* < 0.05 and (**) *p* < 0.01.

**Figure 4:**
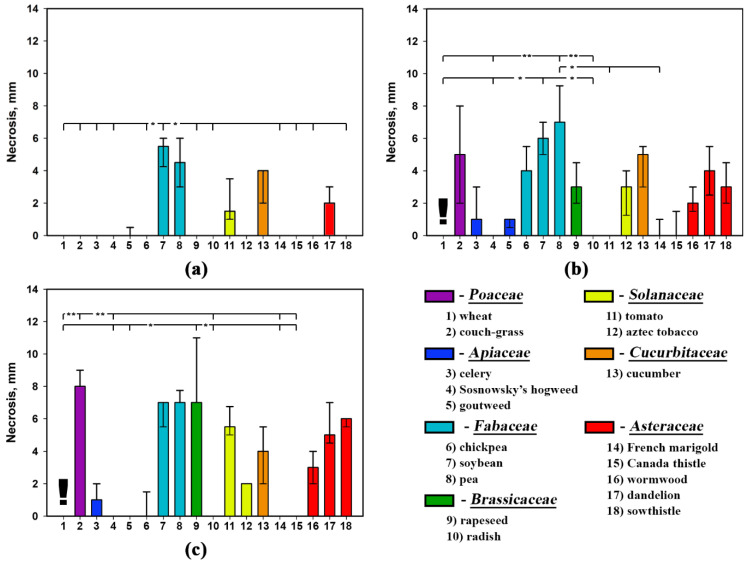
Phytotoxicity of stagonolide K (2 mg/mL) prepared in 5% EtOH assayed on (**a**) non-punctured and (**b**) punctured leaf fragments, and (**c**) supplemented with Hasten™ (0.1% *v*/*v*) on non-punctured leaf fragments of 18 different plant species. Bars represent median values and interquartile ranges. Statistically significant differences between groups were assessed by the Kruskal–Wallis test: (*) *p* < 0.05 and (**) *p* < 0.01. The (!) signs indicate the formation of “green islands”.

**Figure 5:**
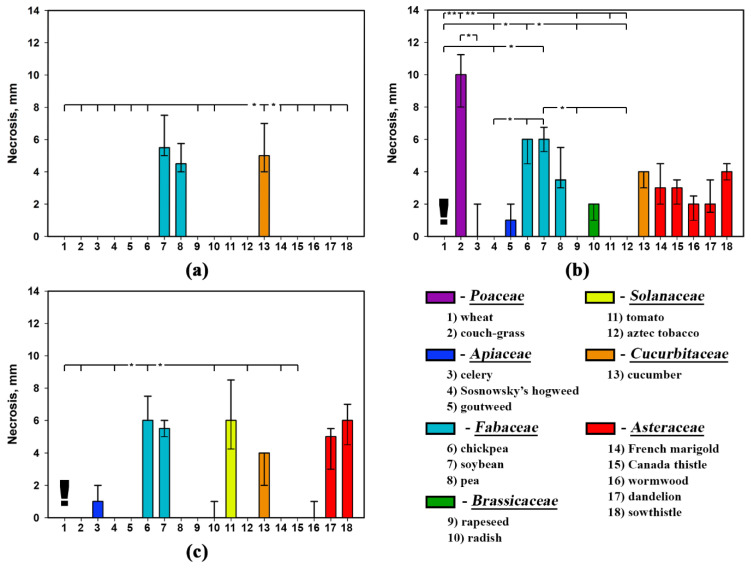
Phytotoxicity of herbarumin I (2 mg/mL) prepared in 5% EtOH assayed on (**a**) non-punctured and (**b**) punctured leaf fragments, and (**c**) supplemented with Hasten™ (0.1% *v*/*v*) on non-punctured leaf fragments of 18 different plant species. Bars represent median values and interquartile ranges. Statistically significant differences between groups were assessed by the Kruskal–Wallis test: (*) *p* < 0.05 and (**) *p* < 0.01. The (!) signs indicate the formation of “green islands”.

Assayed on non-punctured leaf segments, the three nonenolides prepared as the water formulations were not phytotoxic to rapeseed, aztec tobacco, French marigold and perennial sowthistle as well as to *Apiaceae* representatives (Figure 3a, Figure 4a and Figure 5a).

All the toxins affected intact leaf segments of soybean, pea and cucumber to cause necrotic lesions (Figure 3a, Figure 4a and Figure 5a). Stagonolide A was able additionally to penetrate into the non-punctured leaves of wheat, chickpea, radish, tomato, Canada thistle, wormwood and dandelion with development of necrotic lesions (Figure 3a). Additionally, intact tomato and dandelion leaf segments were sensitive to stagonolide K (Figure 4a).

When tested on punctured leaf segments, the water formulation of stagonolide A showed nonselective phytotoxic activity, however, the size of necrotic lesions significantly (*p* < 0.01) varied depended on plant species (Table 4). In particular, wheat, coach-grass, and cucumber were considerably more sensitive to stagonolide A than other plant species (Figure 3b).

Stagonolide A supplemented with Hasten™ (0.1% *v*/*v*) displayed the similar level of non-specific phytotoxic activity with slight differences compared to its application on punctured leaf segments without the adjuvant (Figure 3b,c). The size of necrotic lesions caused by stagonolide A in 0.1% Hasten™ on leaves of the weeds such as Canada thistle, dandelion, and perennial sowthistle was significantly lower than on leaves of some crops as wheat, cucumber and tomato (Figure 3c).

Leaf wounding considerably affected phytotoxicity of both stagonolide K and herbarumin I to widen the number of sensitive plant species up to 12 compared to the treatment of intact leaf segments (five and three species, respectively). The *Fabaceae* species and couch-grass were the most sensitive to both compounds while plants from the *Apiaceae* family were low sensitive to the toxins. There were some differences in selectivity of stagonolide K and herbarumin I: punctured tomato leaves were sensitive to stagonolide K being insensitive to herbarumin I; French marigold and Canada thistle were insensitive to stagonolide K being sensitive to herbarumin I (Figure 4b and Figure 5b).

The addition of Hasten™ (0.1% *v*/*v*) just slightly altered selectivity profile of stagonolide K in the treatment of non-punctured leaf fragments showing 11 of 18 sensitive species compared to the treatment of punctured leaf fragments with water solution of the phytotoxin: intact chickpea leaves were insensitive to stagonolide K supplemented with the adjuvant while tomato leaves became sensitive to this formulation of the phytotoxin (Figure 4b,c).

In contrast to stagonolide A and stagonolide K, the selectivity profile of herbarumin I strongly differed depending on leaf wounding and the adjuvant supplementation. The number of sensitive plant species was restricted to seven species when herbarumin I was applied with Hasten™ in the treatment of non-punctured leaf fragments (Figure 5). Notably, unwounded leaf fragments of couch-grass, pea and several *Asteraceae* representatives were insensitive to herbarumin I prepared in 0.1% Hasten™ solution (Figure 5b,c).

In general, the selectivity profiles of stagonolide K and herbarumin I differed from that of nonselective stagonolide A: these compounds were less phytotoxic than the last and displayed some selectivity (Figure 3, Figure 4 and Figure 5). However, Kruskal–Wallis test supported significant (*p* < 0.001) effect of the plant species on the size of necrotic lesion caused by *S. cirsii* toxins irrespective to leaf wounding or adjuvant supplementation (Table 4).

The factor of leaf wounding/adjuvant supplementation considerably affected sensitivity of the plants to stagonolide A and herbarumin I. It did not alter sensitivity of just three plant species: pea, tomato, and dandelion in the case of the first toxin, and wheat, Sosnowsky’s hogweed, and soybean in the case of the latter. Phytotoxicity of stagonolide K was much less affected by the mentioned factor altering sensitivity of 39% of assayed plant species (Table 5). Notably, the plants from the *Apiaceae* family and radish seem to be from insensitive to low sensitive to stagonolide K or herbarumin I irrespective to leaf wounding/adjuvant supplementation (Figure 3, Figure 4 and Figure 5).

The effect of the toxin structure on the size of necrotic lesions was better pronounced when the water formulation of the *S. cirsii* toxins was assayed on punctured leaf fragments compared to non-punctured ones significantly affecting the sensitivity of 89% and 56% of plant species tested, respectively. When the toxins were formulated in 0.1% Hasten™ the effect of their structure on the size of necrotic lesions was significant for 83% of assayed plant species (Table 6).

**Figure 6:**
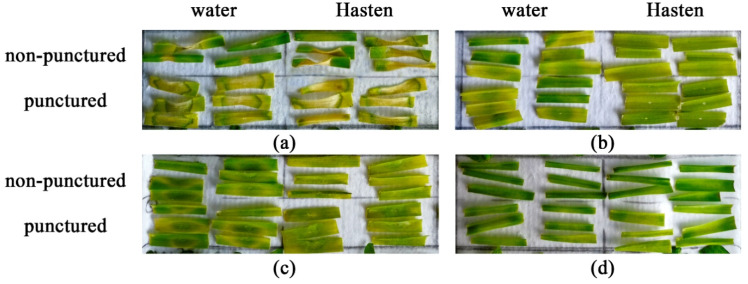
Phytotoxic effect of *S. cirsii* S-47 toxins on wheat leaf segments five days post treatment. Note “green islands” caused by stagonolide K and herbarumin I. (**a**) stagonolide A, (**b**) stagonolide K, (**c**) herbarumin I, (**d**) control.

Notably, it was difficult to assess the effect of stagonolide K and herbarumin I on wheat leaves because the toxin treatment led not to appearing of usual necrotic lesions but to the development of the “green islands” varying from a subtle halo to the clear zone (Figure 6). In the control treatments, neither leaf puncture nor 0.1% Hasten™ caused damage to leaf segments of plants tested.

### 2.3. Contact Herbicidal Activity of S. cirsii Phytotoxins

Spraying of young aerial shoots of *S. arvensis* with a liquid formulation of the phytotoxins of *S. cirsii* at 2 mg/mL (~8.8 mM) in 0.1% Hasten™ resulted in a weak post-emergence herbicidal effect. The fresh biomass weight of toxin-treated sowthistle plants and percentage of necrotic area did not differ from the control treatment (data not shown). Actually, Hasten™ is recommended at a rate 0.5–1% for spray application, so in the further experiment the adjuvant concentration was increased to 0.5% in the herbicidal formulations.

**Figure 7:**
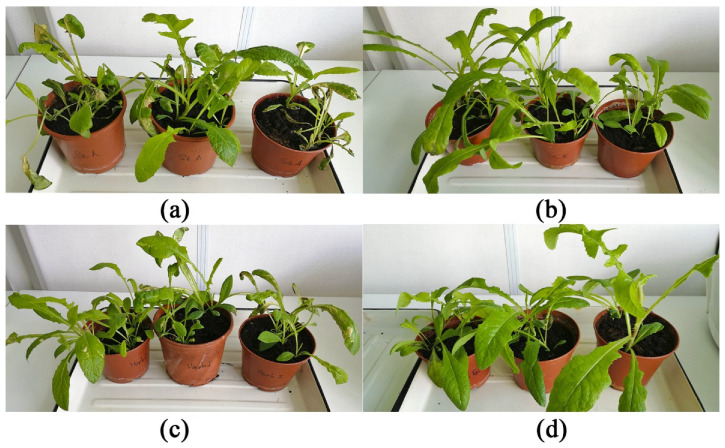
Aerial shoots of perennial sowthistle treated with the *S. cirsii* toxins formulated in 0.5% Hasten™ one week after treatment: (**a**) stagonolide A, (**b**) stagonolide K, (**c**) herbarumin I, (**d**) control.

**Figure 8:**
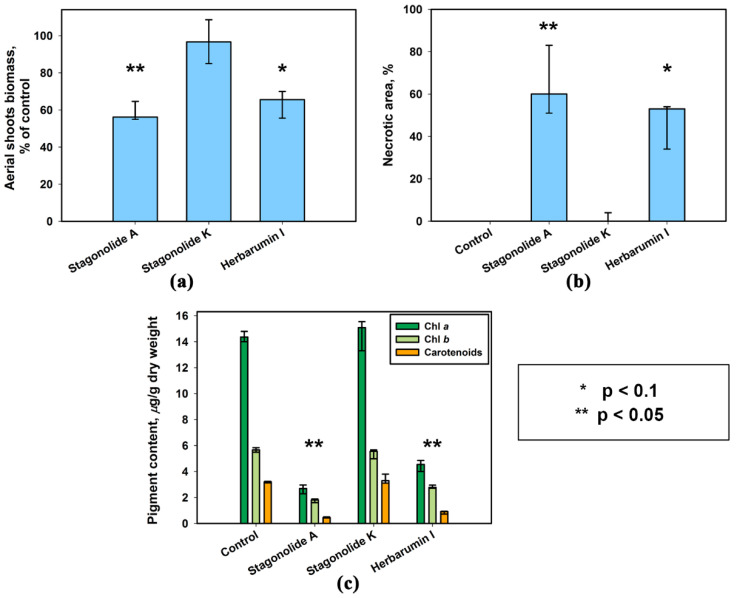
Effect of *S. cirsii* toxins (2 mg/mL) formulated in 0.5% Hasten™ on (**a**) fresh biomass of perennial sowthistle plants, (**b**) percentage of necrotic leaf area and (**c**) photosynthetic pigments content. Bars represent median values and interquartile ranges. Statistically significant differences between groups were assessed by the Kruskal–Wallis test: (*) *p* < 0.05 and (**) *p* < 0.01.

The toxins of *S. cirsii* formulated in 0.5% Hasten™ showed a considerable herbicidal effect on young aerial shoots of *S. arvensis*. In particular, the area of necrotic lesions occupied up to 80% of the leaf surface one week after the treatment of the weed with stagonolide A. The treatment with herbarumin I led to the damage of 50% of the leaf surface. The spraying of the plants with stagonolide K led to the development of single necrotic lesions. Visually, the plants were not damaged when sprayed with 0.5% Hasten™ and water (Figure 7 and Figure 8).

The treatment with stagonolide A and herbarumin I caused a decrease in the fresh biomass weight of the weed plants compared to the control (*p* < 0.05 and *p* < 0.1, respectively) (Figure 8). The effect of the toxins on aerial shoots of *S. arvensis* was manifested as extensive chlorotic and necrotic leaf lesions. The pigment content in toxin-affected leaf tissues was drastically lower than in control leaves (*p* < 0.05). The significant reduction in chlorophylls a and b content was observed in the samples of leaves affected by stagonolide A (81% and 69%, respectively) and herbarumin I (69% and 51% respectively) compared to control treatment. The total carotenoids content in leaves of *S. arvense* followed the same pattern and was found to be maximally reduced (85%) in stagonolide A-treated leaf samples (Figure 8).

## 3. Discussion

The adjuvants in formulations of chemical herbicides are used as wetting agents, penetrants, spreaders, co-solvents, stickers, emulsifiers and others. The practical selection of compatible adjuvant is a complicated task because their positive effect is highly dependent on many factors: the nature of the active ingredient, weed and crop features and application techniques [22,25,33,34]. Our results indicated importance of selection and use of adjuvants in order to increase the leaf penetration and herbicidal activity of natural compounds on the example of three phytotoxic nonenolides.

Searching for the most appropriate adjuvant for *S. cirsii* phytotoxins, we used the commercial products varying in the type and the nature of hydrophilic and lipophilic segments in their molecules (see Section 4.2). Among four adjuvants tested Hasten™ was shown to be most compatible with the fungal phytotoxins (stagonolides A and K, and herbarumin I) allowing them to damage the intact leaf discs of *S. arvensis*. The same adjuvant increased phytotoxic effect of tetramic acid derivative, phaeosphaeride A, on both intact leaf discs and young aerial shoots of Canada thistle (*Cirsium arvense*) [35].

The hydrophilic-lipophilic balance (HLB) is commonly used to suggest the applicability of surfactants (e.g., emulsifiers, detergents) as activator adjuvants [25]. Hasten™, containing non-ionic surfactants and esterified vegetable oil, forms a stable milky dispersion in water which indicates its relatively low HLB value (8–10), whereas other tested adjuvants have HLB number above 16 [25,36]. The oil adjuvants had a higher affinity for the surface waxes of leaves than the surfactants with HLB number 12–20 and ionic surfactants. Moreover, oil-surfactant concentrates can act as co-solvents for herbicides and natural products that have low water solubility. For instance, the herbicidal activity of tenuazonic acid in field experiments was enhanced and stabilized due to the addition of surfactant JN (fatty alcohol polyoxyethylene ether) and lipophilic penetrant laurocapram (1:3, *v*/*v*) [10,37]. Therefore, some oil-based adjuvants, including Hasten™, are effective penetrants striking a balance between herbicide solubility in water carrier and its wax solubility when applied to the plant surface [38]. Surfactants of Hasten™, obviously, assist to retain and improve the contact of the spray droplet on the plant surface.

A few of nonenolides were tested for selectivity. For instance, pinolidoxin (a nonenolide from *Didymella pinodes*, a pathogen of pea) was more toxic to *Fabaceae* than to Canada thistle and perennial sowthistle from the *Asteraceae* family in leaf disc puncture assay [39]. In this study, stagonolide A was proved to be non-selective phytotoxin while stagonolide K and herbarumin I demonstrated some selectivity. Typically, *S. cirsii* toxins caused necrotic leaf lesions on sensitive plants. When assayed on leaf segments of wheat stagonolide K and herbarumin I led to the development of the “green islands” (Figure 6). The similar “green island” effect was observed on cereals leaves treated with pyrenophorol and zinniol [14,40]. In zinniol-treated barley leaf tissues the enhanced chlorophyll retention was demonstrated [40].

Among fungal phytotoxins exhibiting potential for NPDH development, cyclic tetrapeptide tentoxin showed different general response patterns depending on the plant family. The *Brassicaceae* species were all insensitive to tentoxin, whereas other families (*Solanaceae*, *Fabaceae*, *Poaceae*) contained both insensitive and sensitive species [41]. Macrolactone α,ß-dehydrocurvularin caused necrosis on the leaves of 15 of 18 plant species tested and did not cause necrosis on the leaves of two crops (*Zea mays* and *Glycine max*) [15]. A leaf puncture assay of tenuazonic acid on crop and weed species showed that the test-plants demonstrated various sensitivity to the toxin. Different species belonging to *Amaranthaceae*, *Convolvulaceae*, *Asteraceae*, and *Poaceae* demonstrated the sensitivity to tenuazonic acid ranged from moderate to high, while the tested *Malvaceae* and *Solanaceae* plants were tolerant to the toxin [10].

Adjuvants usually improve chemical control of weeds but sometimes they can alter the crop tolerance to herbicides causing species-depended changes of the leaf surface characteristics (trichomes, cuticle and wax structure) [3,38,42]. Similarly, the addition of the adjuvant can alter the selectivity of the formulations of phytotoxins. Indeed, here, we demonstrated some changes in selectivity of three phytotoxic nonenolides when applied in formulations with 0.1% Hasten™ that is important for their future development as NPDHs. The analysis of selectivity profile of the *S. cirsii* toxin supposes some prospects of stagonolide K and herbarumin I for weed control in radish and *Apiaceae* crops, while stagonolide A can be used as a nonselective herbicide.

The efficacy of post-emergent herbicides can be influenced by the type and concentration of an adjuvant included in the formulation. In order to find the most compatible adjuvant for *S. cirsii* herbicidal compounds, we have conducted a widely used leaf disc-puncture bioassay. In this bioassay, a 10-µL droplet is applied on wounded leaf surface with following incubation in a wet chamber, allowing us to evaluate the adjuvant effect on the solubility of the compounds and its phytotoxicity. However, such a bioassay obviously does not consider the effect of the adjuvant on many other parameters of the herbicidal formulation, which are important in the practice, such as the droplet formation and plant surface coverage, the drift and evaporation of spraying mixture, etc. [25,43,44]. It can partially explain the differences in sensitivity of leaf segments and whole plants to three phytotoxic nonenolides in 0.1% Hasten™. The increase in Hasten™ concentration to 0.5% led to the improve of post-emergence herbicidal activity of *S. cirsii* toxins (Figure 3c, Figure 4c, Figure 5c and Figure 8). Therefore, optimization of herbicidal formulations of phytotoxins and Hasten™ should be performed on whole plants using special spray equipment. The spraying properties of herbicidal formulations supplemented with the oil-based adjuvants are largely affected by the type of emulsifier (to a greater extent) and fatty acid composition (to a lesser extent) [38]. Further selection of optimal oil-based adjuvants seems to be a promising way to increase the herbicidal activity of the *S. cirsii* phytotoxins and other hydrophobic natural compounds.

The photosynthetic pigment content of the leaves provides valuable insight into the physiological performance of plants including *Sonchus* spp. It is often used as an indicator of senescence, stress or damage to the photosynthetic apparatus. For instance, some herbicides, as well as heavy metals, drought, and ozone may affect the pigment content in *Sonchus* leaves [45,46,47,48]. Many fungal phytotoxins directly (for instance, macrocidin A, tentoxin or tenuazonic acid) [9,49,50] or possibly indirectly (e.g., dehydrocurvularin, pyrenophorin, cytochalasin E or fusaric acid) [15,51,52,53] induce malfunctioning of photosynthetic machinery followed by cell death. Our results showed stagonolide A and herbarumin I to decrease chlorophyll and carotenoid content in toxin-treated leaf tissues confirming their adverse effect on photosynthesis. For stagonolide A, this observation is consistent with the results of Berestetskiy et al. (2008) [54] showed that stagonolide A decreased absorption at a wavelength of 450 nm in toxin-treated leaf discs of Canada thistle, which may correlate with the content of chlorophylls and/or carotenoids. Further elucidation of the action mechanisms of both nonenolides may be a perspective step in the development of a novel nature-derived herbicide [55].

## 4. Materials and Methods

### 4.1. Fungal Strain and Toxin Production

The strain S-47 of *S. cirsii* used in this study has been deposited in the collection of the All-Russian Institute of Plant Protection (Pushkin, Saint-Petersburg, Russia). The submerged fermentation was conducted in a 7 L fermenter (Applikon Biotechnology, Delft, The Netherlands) containing 5 L of modified Czapek medium. The solid-state fermentation was carried out in a 1500 mL flat culture flask. The fungus was grown on autoclaved millet at 12 h photoperiod (day temperature 24 °C, night temperature 20 °C) for 2 weeks [26,32]. The fermentation of the fungus as well as extraction and purification of stagonolides A, K and herbarumin I were performed as described by Dalinova et al., 2019 [32].

### 4.2. Effect of Adjuvants on Phytotoxic Activity

Three different non-ionic adjuvants, Trend^®^-90 (isodecyl alcohol ethoxylate, Du Pont, Geneva, Switzerland), Tween^®^-20 (polyoxyethylene sorbitol ester, Croda Crop., Snaith, UK), and Hasten™ (ethyl and methyl esters of vegetable oil, Victorian Chemicals, Coolaroo, Australia), and one anionic adjuvant, Biopower^®^ (sodium lauryl sulphate, Bayer CropScience Limited, Cambridge, UK) were used in this bioassay. The solutions of the adjuvants were prepared in distilled water in the following concentrations (*v*/*v*): 0.1% Tween^®^-20, 0.1% Biopower^®^, 0.01% Trend^®^-90 and 0.1% Hasten™. The samples of stagonolides A, K and herbarumin I (0.4 mg each) were dissolved in 10 μL of EtOH and adjusted to the volume of 200 μL with one of the adjuvant solutions or water. The final concentration of ethanol was 5% (*v*/*v*), the concentration of the tested toxins was 2 mg/mL (*w*/*v*) (~8.8 mM). Solutions of the tested adjuvants in 5% EtOH were used as control treatments. The phytotoxic effect of the compounds was assessed on non-punctured and punctured leaf discs of *S. arvensis*. Discs 1 cm in diameter were cut with a cork drill from the leaves of 3–5-week-old sowthistle plants and placed in a wet chamber. After that, half of them were punctured in the center with a dissecting needle, and 10 μL of test-solution was applied to the central area of each disc. The details of leaf puncture bioassay are given in Berestetskiy et al., 2010 [56] and Poluektova et al., 2018 [35]. The diameter of the necrotic lesions was measured 120 h after treatment. Ten replicate leaf discs were used for each treatment.

### 4.3. Phytotoxic Selectivity of S. cirsii Toxins

The following crops and weeds belonging to different families were used as test-plants for the assay: *Poaceae*: common wheat (*Triticum aestivum* L.), couch-grass (*Elytrigia repens* (L.) Nevski); *Apiaceae:* celery (*Apium graveolens* L.), goutweed (*Aegopodium podagraria* L.), Sosnowsky’s hogweed (*Heracleum sosnowskyi* Manden.); *Fabaceae*: chickpea (*Cicer arietinum* L.), soybean (*Glycine max* (L.) Merr.), pea (*Pisum sativum* L.); *Brassicaceae*: radish (*Raphanus raphanistrum* L.), rapeseed (*Brassica napus* L.); *Solanaceae*: tomato (*Lycopersicon esculentum* L.), aztec tobacco (*Nicotiana rustica* L.); *Cucurbitaceae*: cucumber (*Cucumis sativus* L.); *Asteraceae*: French marigold (*Tagetes patula* L.), Canada thistle (*Cirsium arvense* (L.) Scop.), common dandelion (*Taraxacum officinale* (L.) Weber ex F.H. Wigg), wormwood (*Artemisia absinthium* L.) and sowthistle (*Sonchus arvensis* L.). The samples of tested toxins were dissolved in ethanol and then diluted with water or 0.1% Hasten™ (*v*/*v*), as described above, to the concentration of 2 mg/mL (~8.8 mM). Punctured and non-punctured leaf segments (leaf cuttings (2 cm long) for monocotyledons and leaf discs (1 cm in diameter) for dicotyledons) were treated with 10 μL droplets of toxin solutions. At least eight replicate leaf segments were used for each treatment. The treated leaf parts were incubated in wet chamber at 24 °C for 120 h. The phytotoxic activity was determined as the length or diameter of necrotic lesions (for monocotyledons and dicotyledons respectively) 120 h after treatment.

### 4.4. Herbicidal Activity of Phytotoxins

The herbicidal effect of the most phytotoxic formulations of stagonolides A, K and herbarumin I was evaluated on plants of *S. arvensis* at the rosette stage. The underground shoots of the weed (cuttings 5 cm long) were planted in pots with soil mixture and have been incubated at 24 °C and 12-h photoperiod for 4 weeks. The samples (30 mg) of phytotoxins were dissolved in 750 μL of ethanol. These solutions were diluted to the volume of 15 mL with 0.1% and 0.5% Hasten™ (*v*/*v*). The final concentrations of the toxins and ethanol were 2 mg/mL (~8.8 mM) and 5% (*v*/*v*), respectively. The formulations of stagonolides A, K, and herbarumin I were sprayed onto the plants with a hand atomizer (3 mL per plant, 4 replicate pots per treatment). The 5% ethanol in 0.1% and 0.5% Hasten™, respectively, was used as a control treatment. The sprayed plants were incubated at 24 °C for a 12-h photoperiod. Herbicidal efficacy was assessed visually 48 h and one week after treatment. The aerial shoots of all the test-plants were cut after one week of incubation and weighted. The herbicidal efficacy of the formulation of *S. cirsii* toxins was assessed as the percentage of necrotic leaf area and fresh weight biomass of *S. arvensis* compared to the control pots.

### 4.5. Quantification of Photosynthetic Pigments

To analyze the effect of toxins on pigment content, the leaf samples (20–30 mg) were cut from control and toxin damaged plants. Photosynthetic pigments were extracted from leaf samples with 100% acetone as described by Lichtenthaler and Buschmann, 2001 [57]. The dry weight was used as a reference system. To determine the water content in control and damaged leaves, further weighted samples of plant material were placed in an aluminum dishes, dried for 2 h at 100 °C, and weighted again. Pigment contents were calculated using the following formulae:

Chlorophyll a (c_a_, μg/mL) = 11.24 A_662_ − 2.04 A_645_

Chlorophyll b (c_b_, μg/mL) = 20.13 A_645_ − 4.19 A_662_

Carotenoids (c_x+c_, μg/mL) = (1000 A_470_ − 1.90 c_a_ − 63.14 c_b_)/214

Chlorophyll a (mg/g dw) = c_a_ ∗ V/W

Chlorophyll b (mg/g dw) = c_b_ ∗ V/W

Carotenoids (mg/g dw) = c_x+c_ ∗ V/W,

where V = volume of solvent (5 mL), and W = dry weight of leaf sample [57].

### 4.6. Statistical Data Analysis

The results of bioassays were subjected to the non-parametric Kruskal–Wallis test for determination of significant differences between variants at *p* = 0.05 because the data in the compared groups are not normally distributed. The statistical analysis was performed using Statistica 8.0 (StatSoft, Tusla, OK, USA).

## 5. Conclusions

The adjuvant Hasten™ (0.1%, *v*/*v*) significantly increased phytotoxic activity of stagonolides A and K, and herbarumin I on intact leaf discs of *S. arvensis*. Stagonolide A was shown to be nonselective toxin, while stagonolide K and herbarumin I demonstrated selective phytotoxic action. The selectivity profile of the two latter compounds combined with Hasten™ was changed slightly to show their prospects for weed control in radish and some *Apiaceae* crops. When tested on whole plants of perennial sowthistle, the toxins (0.2% solution (*w*/*v*) in 0.5% Hasten™ (*v*/*v*)) displayed moderate contact herbicidal activity causing the leaf damage, decreased content of photosynthetic pigments, and the loss of aerial shoots biomass. Our results demonstrated that stagonolide A and herbarumin I supplemented with Hasten™ are promising candidates for development of NPDH. Moreover, the study confirms the potential of Hasten™ to increase the herbicidal efficacy of natural phytotoxins. Further investigations are necessary to screen oil-based adjuvants and to select their concentration for more effective foliar application of *S. cirsii* and other phytotoxins.

## Figures and Tables

**Table 1:** Effect of adjuvant (dF = 4) on differences in phytotoxicity of three nonenolides to punctured and non-punctured leaf discs of perennial sowthistle by Kruskal–Wallis test.

Leaf Wounding	Phytotoxin		
	Stagonolide A	Stagonolide K	Herbarumin I
punctured	H = 6.34, *p* = 0.175	H = 12.58, *p* = 0.014 *	H = 9.76, *p* = 0.045 *
non-punctured	H = 16.81, *p* = 0.002 *	H = 22.85, *p* = 0.000 *	H = 21.15, *p* = 0.000 *

* *p* < 0.05.

**Table 2:** Effect of leaf wounding (dF = 1) on differences in phytotoxicity of three nonenolides formulated in different adjuvants to leaf discs of perennial sowthistle by Kruskal–Wallis test.

Adjuvant	Phytotoxin		
	Stagonolide A	Stagonolide K	Herbarumin I
Tween^®^-20	H = 7.37, *p* = 0.007 *	H = 4.95, *p* = 0.026 *	H = 6.53, *p* = 0.012 *
BioPower^®^	H = 6.02, *p* = 0.014 *	H = 6.88, *p* = 0.009 *	H = 3.58, *p* = 0.059
Trend^®^ 90	H = 2.80, *p* = 0.094	H = 0.11, *p* = 0.742	H = 8.47, *p* = 0.004 *
Hasten™	H = 0.11, *p* = 0.737	H = 1.88, *p* = 0.171	H = 0.06, *p* = 0.803
Water	H = 9.54, *p* = 0.002 *	H = 7.21, *p* = 0.007 *	H = 9.66, *p* = 0.001 *

* *p* < 0.05.

**Table 3:** Effect of the toxin structure (dF = 2) on differences in phytotoxicity of their formulations in different adjuvants to punctured and non-punctured leaf discs of perennial sowthistle by Kruskal–Wallis test.

Leaf Wounding	Adjuvant				
	Tween^®^-20	BioPower^®^	Trend^®^ 90	Hasten™	Water
punctured	H = 5.78, *p* = 0.056	H = 10.28, *p* = 0.006 *	H = 2.25, *p* = 0.325	H = 1.43, *p* = 0.490	H = 11.19, *p* = 0.037 *
non-punctured	H = 6.47, *p* = 0.039 *	H = 8.72, *p* = 0.013 *	H = 9.21, *p* = 0.010 *	H = 0.26, *p* = 0.878	H = 0.00, *p* = 1.000

* *p* < 0.05.

**Table 4:** Effect of plant species (dF = 17) on differences in phytotoxicity of three nonenolides in different treatments (formulation/wounding) of leaf fragments by Kruskal–Wallis test.

Formulation/Wounding	Phytotoxin		
	Stagonolide A	Stagonolide K	Herbarumin I
Water/ non-punctured	H = 77.61, *p* = 0.000 *	H = 82.39, *p* = 0.000 *	H = 82.73, *p* = 0.000 *
Water/ punctured	H = 67.55, *p* = 0.000 *	H = 73.33, *p* = 0.000 *	H = 77.34, *p* = 0.000 *
0.1% Hasten™/ non-punctured	H = 71.01, *p* = 0.000 *	H = 82.40, *p* = 0.000 *	H = 80.43, *p* = 0.000 *

* *p* < 0.05.

**Table 5:** Effect of the treatment (formulation/wounding, dF = 2) on differences in phytotoxicity of three nonenolides to leaf fragments of 18 plant species by Kruskal–Wallis test.

Plant Species	Phytotoxin		
	Stagonolide A	Stagonolide K	Herbarumin I
wheat	H = 14.60, *p* = 0.002 *	H = 0.00, *p* = 1.000	H = 0.00, *p* = 1.000
couch-grass	H = 15.41, *p* = 0.002 *	H = 12.63, *p* = 0.006 *	H = 16.00, *p* = 0.001 *
celery	H = 15.59, *p* = 0.001 *	H = 16.52, *p* = 0.001 *	H = 15.26, *p* = 0.002 *
Sosnowsky’s hogweed	H = 12.55, *p* = 0.006 *	H = 0.00, *p* = 1.000	H = 0.00, *p* = 1.000
goutweed	H = 14.48, *p* = 0.002 *	H = 6.65, *p* = 0.084	H = 12.44, *p* = 0.006 *
chickpea	H = 8.36, *p* = 0.039 *	H = 15.71, *p* = 0.001 *	H = 14.89, *p* = 0.002 *
soybean	H = 12.39, *p* = 0.006 *	H = 4.69, *p* = 0.196	H = 1.67, *p* = 0.644
pea	H = 4.00, *p* = 0.261	H = 8.57, *p* = 0.036 *	H = 12.46, *p* = 0.006 *
rapeseed	H = 15.16, *p* = 0.002 *	H = 16.76, *p* = 0.001 *	H = 6.32, *p* = 0.097
radish	H = 9.51, *p* = 0.023 *	H = 0.00, *p* = 1.000	H = 11.29, *p* = 0.010 *
tomato	H = 5.91, *p* = 0.116	H = 13.45, *p* = 0.004 *	H = 13.26, *p* = 0.004 *
aztec tobacco	H = 17.15, *p* = 0.001 *	H = 12.55, *p* = 0.006 *	H = 18.55, *p* = 0.000 *
cucumber	H = 9.93, *p* = 0.019 *	H = 6.17, *p* = 0.104	H = 12.42, *p* = 0.006 *
French marigold	H = 10.97, *p* = 0.012 *	H = 4.75, *p* = 0.191	H = 16.75, *p* = 0.001 *
Canada thistle	H = 7.70, *p* = 0.053	H = 14.35, *p* = 0.003 *	H = 16.65, *p* = 0.001 *
wormwood	H = 10.44, *p* = 0.015 *	H = 15.64, *p* = 0.001 *	H = 15.65, *p* = 0.001 *
dandelion	H = 3.60, *p* = 0.309	H = 5.98, *p* = 0.112	H = 13.80, *p* = 0.003 *
perennial sowthistle	H = 11.53, *p* = 0.009 *	H = 16.62, *p* = 0.001 *	H = 14.94, *p* = 0.002 *

* *p* < 0.05.

**Table 6:** Effect of the toxin structure (dF = 2) on differences in their phytotoxicity in different treatments (formulation/wounding) of leaf fragments of 18 plant species by Kruskal–Wallis test.

Plant Species	Formulation/Wounding		
	Water/ Non-Punctured	Water/ Punctured	0.1% Hasten™/ Non-Punctured
wheat	H = 13.32, *p* = 0.001 *	H = 13.43, *p* = 0.001 *	H = 13.46, *p* = 0.001 *
couch-grass	H = 10.22, *p* = 0.006 *	H = 5.58, *p* = 0.061	H = 13.23, *p* = 0.001 *
celery	H = 0.00, *p* = 1.000	H = 5.41, *p* = 0.067	H = 4.67, *p* = 0.097
Sosnowsky’s hogweed	H = 0.00, *p* = 1.000	H = 13.43, *p* = 0.001 *	H = 13.46, *p* = 0.001 *
goutweed	H = 2.00, *p* = 0.368	H = 9.20, *p* = 0.010 *	H = 13.43, *p* = 0.001 *
chickpea	H = 13.36, *p* = 0.001 *	H = 1.07, *p* = 0.585	H = 12.73, *p* = 0.002 *
soybean	H = 7.83, *p* = 0.020 *	H = 5.37, *p* = 0.068	H = 2.59, *p* = 0.274
pea	H = 2.14, *p* = 0.342	H = 8.38, *p* = 0.015 *	H = 10.51, *p* = 0.005 *
rapeseed	H = 0.00, *p* = 1.000	H = 10.18, *p* = 0.006 *	H = 13.21, *p* = 0.001 *
radish	H = 12.63, *p* = 0.002 *	H = 13.13, *p* = 0.001 *	H = 11.69, *p* = 0.003 *
tomato	H = 9.40, *p* = 0.009 *	H = 10.51, *p* = 0.005 *	H = 2.36, *p* = 0.307
aztec tobacco	H = 0.00, *p* = 1.000	H = 13.08, *p* = 0.001 *	H = 13.51, *p* = 0.001 *
cucumber	H = 5.03, *p* = 0.080	H = 9.75, *p* = 0.008 *	H = 10.00, *p* = 0.007 *
French marigold	H = 2.00, *p* = 0.368	H = 9.90, *p* = 0.007 *	H = 13.32, *p* = 0.001 *
Canada thistle	H = 13.43, *p* = 0.001 *	H = 11.66, *p* = 0.003 *	H = 13.32, *p* = 0.001 *
wormwood	H = 13.43, *p* = 0.001 *	H = 0.89, *p* = 0.640	H = 9.80, *p* = 0.008 *
dandelion	H = 10.22, *p* = 0.006 *	H = 3.54, *p* = 0.170	H = 2.62, *p* = 0.270
sowthistle	H = 0.00, *p* = 1.000	H = 8.80, *p* = 0.012 *	H = 0.64, *p* = 0.725

* *p* < 0.05.
